# Dietary pulp from *Fructus Schisandra Chinensis* supplementation reduces serum/hepatic lipid and hepatic glucose levels in mice fed a normal or high cholesterol/bile salt diet

**DOI:** 10.1186/1476-511X-13-46

**Published:** 2014-03-12

**Authors:** Nan Sun, Si-Yuan Pan, Yi Zhang, Xiao-Yan Wang, Pei-Li Zhu, Zhu-Sheng Chu, Zhi-Ling Yu, Shu-Feng Zhou, Kam-Ming Ko

**Affiliations:** 1 Department of Pharmacology, School of Chinese Materia Medica, Beijing University of Chinese Medicine, Beijing 100102, China; 2 School of Chinese Medicine, Hong Kong Baptist University, Hong Kong, China; 3 Department of Pharmaceutical Sciences, College of Pharmacy, University of South Florida, Florida 33612, USA; 4 Division of Life Science, Hong Kong University of Science & Technology, Hong Kong, China

**Keywords:** *Fructus Schisandrae Chinensis*, Bicyclol, Fatty liver, Hypercholesterolemia, Epididymal fat, Glucose

## Abstract

**Background:**

Recently, it has been found that *Fructus Schisandra Chinensis* (FSC), a Chinese herbal medicine, and its related compounds have a profound impact on lipid metabolism process. FSC can be divided into two parts, i.e., seed and pulp. The current study aimed to examine the effect of aqueous extracts of FSC pulp (AqFSC-P) on serum/hepatic lipid and glucose levels in mice fed with a normal diet (ND) or a high cholesterol/bile salt diet (HCBD).

**Methods:**

The AqFSC-P used in the present study was fractionated into supernatant (SAqFSC-P) and precipitate (PAqFSC-P) separated by centrifugation. Male ICR mice were fed with ND or HCBD, without or with supplementation of 1%, 3%, or 9% (w/w) SAqFSC-P or PAqFSC-P for 10 days. Biomarkers were determined according to the manufacturer’s instruction.

**Results:**

Supplementation with SAqFSC-P or PAqFSC-P significantly reduced serum and hepatic triglyceride levels (approximately 40%) in ND- and/or HCBD-fed mice. The supplementation with SAqFSC-P or PAqFSC-P reduced hepatic total cholesterol levels (by 27 - 46%) in HCBD-fed mice. Supplementation with SAqFSC-P or PAqFSC-P markedly lowered hepatic glucose levels (by 13 - 30%) in ND- and HCBD-fed mice. SAqFSC-P decreased serum alanine aminotransferase (ALT) activity, but PAqFSC-P increased hepatic protein contents in ND-fed mice. Bicylol, as a positive control, reduced ALT activity. In addition, mice supplemented with FSC-P or bicylol showed a smaller body weight gain and adipose tissue mass as compared to the respective un-supplemented ND- or HCBD-fed mice.

**Conclusion:**

The results indicate that SAqFSC-P and PAqFSC-P produce hepatic lipid- and glucose-lowering as well as serum TG-lowering effects in hypercholesterolemic mice. FSC pulp may provide a safe alternative for the management of fatty liver and/or lipid disorders in humans.

## Background

A growing body of evidence has shown that hyperlipidemia, regardless of cause, is an important factor involved in the development of a wide range of human diseases, including coronary heart disease (CHD) [1,2], nonalcoholic fatty liver disease (NAFLD) [3] and metabolic syndrome [4]. Therefore, the searching for therapeutic intervention aimed at lowering lipid levels in blood and the liver has been an area of intensive research. While lipid-lowering drugs can be used to lower the elevated levels of lipids in patients with dyslipidemia [5], there is still no effective drug for the treatment of NAFLD, which is manifested as deposition of fat in the liver in patients without an excessive consumption of alcohol. The commonly prescribed lipid-lowering drugs can cause many potential adverse/side effects, such as myopathies, renal impairment, hepatic injury, and or pancreatitis [6,7]. Therefore, it is of clinical interest to manage hyperlipidemia with naturally-occurring ingredients such as coenzyme Q10 [8], phytosterols [9], unsaturated fatty acids [10,11], probiotics [12], as well as herbs [13,14].

*Fructus Schisandra Chinensis* (FSC, Wu-wei-zi in Chinese), a traditional Chinese tonic herb, has been used for thousands of years in China. In generally, FSC can be divided into two parts — seed and pulp. Previous studies have shown that FSC extract (both seed and pulp) [15,16] or its active ingredient schisandrin B [17] and other related compounds bicyclol [18] and bifendate [19] can reduce hepatic triglyceride (TG) and total cholesterol (TC) levels in mice with hypercholesterolemia induced by high-fat diet containing cholesterol/bile salt. In this study, we endeavored to investigate whether the two fractions of the aqueous extract of FSC pulp (AqFSC-P), namely, supernatant (SAqFSC-P) and precipitate (PAqFSC-P), can decrease serum and hepatic lipid/glucose (GLU) levels in normal and hypercholesterolemic mice. Serum or hepatic lipids including TG, TC, low-density lipoprotein (LDL), high-density lipoprotein (HDL), as well as HDL/LDL, LDL/HDL, and non-HDL (N-HDL) were measured. Bicyclol, a synthetic derivative of dibenzocyclooctadiene lignans from the FSC, was used as positive control for comparison. The objective of this study is to establish the pharmacological basis for the potential use of FSC-P for the treatment of the patients with hypertriglyceridemia, fatty liver disease, and obesity. As the residue from the manufacture process of FSC is often treated as waste, the effects of supplementation with the precipitate of AqFSC-P on lipids were compared with those of the supernatant of AqFSC-P.

## Results

### Serum lipid levels

**Figure 1:**
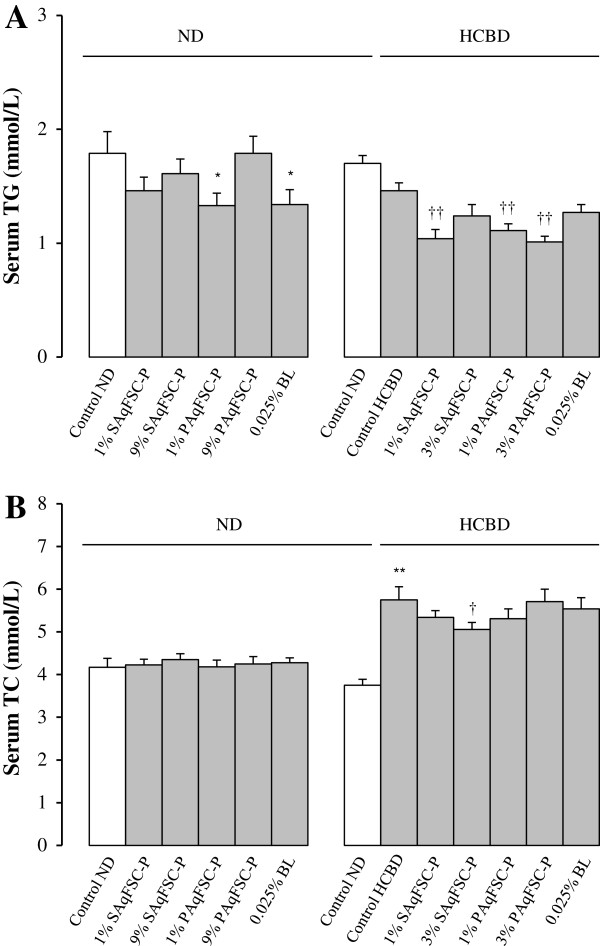
**Effects of dietary supplementation with SAqFSC-P, PAqFSC-P, or bicyclol on serum TG and TC levels in normal and hypercholesterolemic mice.** Mice were fed with normal diet (ND) or high cholesterol/bile salt (1%/0.3%, w/w) diet (HCBD) without and with the supplementation with supernatant/precipitate of aqueous extract of pulp from *Fructus Schisandrae Chinesis* [namely, SAqFSC-P/PAqFSC-P (1%, 3%, or 9%, w/w)] or bicyclol (BL, 0.025%, w/w) for 10 days. The concentrations of SAqFSC-P/PAqFSC-P were estimated on the basis of crude herbal material. Serum triglyceride (TG) and total cholesterol (TC) levels (as indicated in Figure **A** and **B**, respectively) were measured. Value given are the means ± S.E.M., with n = 10. ^*^P < 0.05, ^**^P < 0.01 vs. control ND; ^†^*P* < 0.05, ^††^*P* < 0.01 vs. control HCBD, using a one-way ANOVA followed by Dunnett’s multiple comparisons test or post-hoc analysis.

Supplementation with SAqFSC-P and PAqFSC-P did not affect serum TG and TC levels in mice fed with ND, except for PAqFSC-P at a dose of 1% which showed a significant decrease in serum TG level (Figure 1). While HCBD feeding slightly decreased serum TG level, both SAqFSC-P and PAqFSC-P supplementations further decreased serum TG level (by 23 - 30%) in HCBD-fed (i.e., hypercholesterolemic) mice (Figure 1A). HCBD feeding markedly increased serum TC level by 53% in mice (Figure 1B), but PAqFSC-P supplementations did not affect serum TC levels in HCBD-fed mice. SAqFSC-P (3%) decreased serum TC levels in hypercholesterolemic mice. Bicyclol supplementation did not affect serum TG and TC levels in both ND- and HCBD-fed mice. In addition, although HCBD intake did not affect the serum HDL levels, it increased serum LDL level, LDL/HDL ratio, and N-HDL level by 440%, 500%, and 375%, respectively, and decreased the HDL/LDL ratio by 83%. FSC-P treatment did not affect serum HDL and LDL level in ND-fed mice. However, supplementation with PAqFSC-P (1% or 3%) attenuated N-HDL levels by 15 and 22%, respectively, in HCBD-fed mice, when compared the un-supplemented HCBD-fed mice (Table 1).

**Table 1:** Effects of dietary supplementation with SAqFSC-P, PAqFSC-P, or bicyclol on serum lipoprotein profiles in normal and hypercholesterolemic mice

**Groups**	**Drug concentration (%, w/w)**	**HDL (mmol/L)**	**LDL (mmol/L)**	**HDL/LDL (mmol/L)**	**LDL/HDL (mmol/L)**	**N-HDL (mmol/L)**
***Normal mice***						
Control ND	-	3.94 ± 0.21	0.71 ± 0.04	5.82 ± 0.34	0.18 ± 0.01	0.11 ± 0.11
SAqFSC-P/ND	1	3.95 ± 0.09	0.67 ± 0.03	6.02 ± 0.28	0.17 ± 0.01	0.23 ± 0.13
	9	4.00 ± 0.11	0.63 ± 0.04	6.47 ± 0.30	0.16 ± 0.01	0.25 ± 0.09
PAqFSC-P/ND	1	4.00 ± 0.15	0.63 ± 0.03	6.20 ± 0.36	0.16 ± 0.01	0.23 ± 0.06
	9	4.18 ± 0.17	0.63 ± 0.03	6.46 ± 0.29	0.16 ± 0.01	0.17 ± 0.06
Bicyclol/ND	0.025	4.17 ± 0.16	0.70 ± 0.04	6.21 ± 0.48	0.17 ± 0.02	0.10 ± 0.07
***Hypercholester-olemic mice***						
Control ND	-	3.10 ± 0.12	0.20 ± 0.02	16.65 ± 1.30	0.06 ± 0.01	0.64 ± 0.03
Control HCBD	-	3.02 ± 0.12	1.08 ± 0.07^**a**^	2.86 ± 0.17^**a**^	0.36 ± 0.02^**a**^	2.54 ± 0.14^**a**^
SAqFSC-P/HCBD	1	3.15 ± 0.15	1.08 ± 0.06	3.29 ± 0.16	0.31 ± 0.02	2.17 ± 0.12
	3	3.20 ± 0.12	1.22 ± 0.09	2.90 ± 0.14	0.35 ± 0.02	2.51 ± 0.19
PAqFSC-P/HCBD	1	3.16 ± 0.10	1.09 ± 0.06	2.72 ± 0.17	0.37 ± 0.02	2.18 ± 0.10^**b**^
	3	3.08 ± 0.10	0.93 ± 0.05	3.30 ± 0.21	0.35 ± 0.02	1.98 ± 0.11^**b**^
Bicyclol/HCBD	0.025	3.27 ± 0.14	1.02 ± 0.07	3.41 ± 0.23	0.31 ± 0.02	2.28 ± 0.13

Experimental details were described in Figure 1. Mice were fed with ND or HCBD containing SAqFSC-P, PAqFSC-P, or bicyclol at the indicated concentration for 10 days. Serum low-density lipoprotein (LDL), high-density lipoprotein (HDL), HDL/LDL, LDL/HDL, and N-HDL levels were then measured. Mouse model of hypercholesterolemia was generated by HCBD. Values given are the means ± S.E.M., with n = 10. ^**a**^*P* < 0.01 vs. control ND; ^**b**^*P* < 0.05 vs. control HCBD, using a one-way ANOVA follow by Dunnett’s multiple comparisons test or post-hoc analysis.

### Hepatic lipid levels

**Figure 2:**
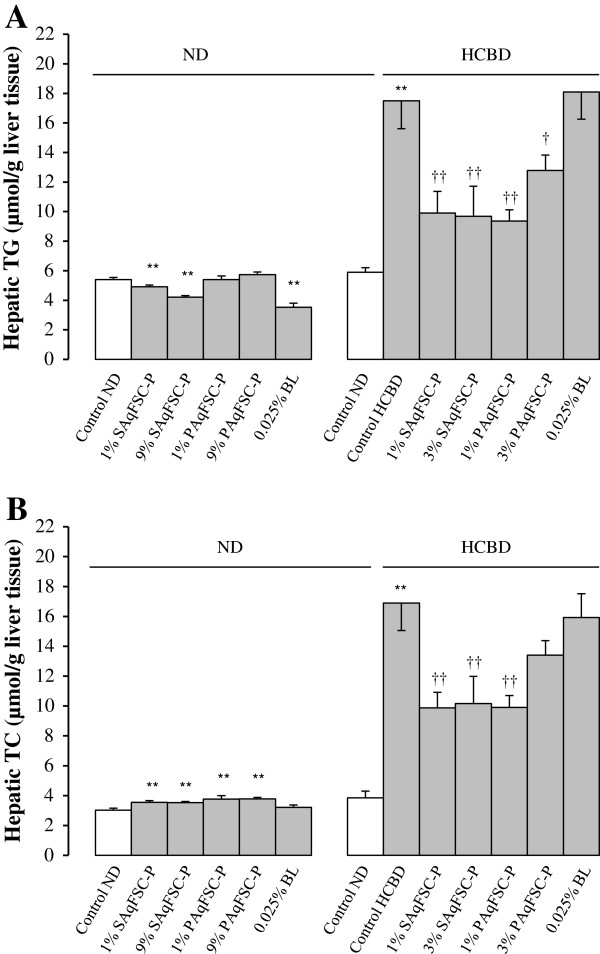
**Effects of dietary supplementation with SAqFSC-P, PAqFSC-P, or bicyclol on hepatic TG and TC contents in normal and hypercholesterolemic mice.** Mice were fed with ND or HCBD without and with supplementation with SAqFSC-P, PAqFSC-P, or BL at the indicated concentration for 10 days. Experimental details were described in Figure 1. Hepatic TG (Figure **A**) and TC (Figure **B**) levels were measured. Value given are the means ± S.E.M., with n = 10. ^**^P < 0.01 vs. control ND; ^†^*P* < 0.05, ^††^*P* < 0.01 vs. control HCBD, using a one-way ANOVA followed by Dunnett’s multiple comparisons test or post-hoc analysis.

Mice fed ND supplemented with 1% or 9% SAqFSC-P for 10 days showed a significant decrease in hepatic TG content by 9 and 22%, respectively (Figure 2A). Supplementation with SAqFSC-P or PAqFSC-P at doses of 1% and 9% increased hepatic TC contents (by 6 - 18%) in ND-fed mice (Figure 2B). Feeding mice with HCBD markedly increased hepatic TG and TC contents (by 196 and 339%, respectively), when compared with those of mice fed with ND. Supplementation with SAqFSC-P and PAqFSC-P at 1% or 3% decreased hepatic TC and TG contents (by 27 - 46% and 40 - 42%, respectively) in HCBD-fed mice. However, 3% PAqFSC-P did not decrease hepatic TC in hypercholesterolemic mice. While 0.025% bicyclol supplementation reduced hepatic TG content (by 32%) in ND-fed mice, it did not affect hepatic TG and TC contents in HCBD-fed mice.

### Serum and hepatic glucose levels

**Figure 3:**
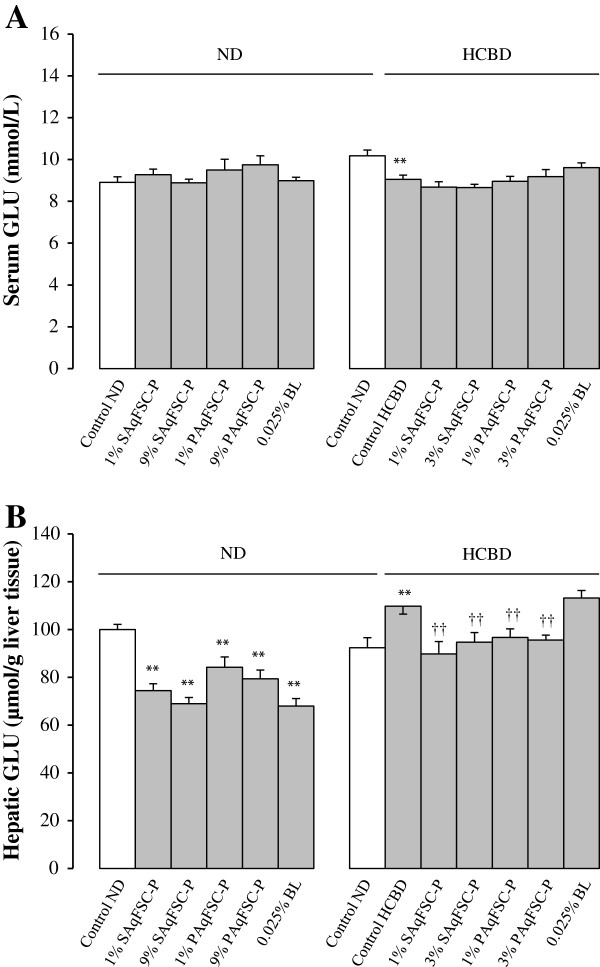
**Effects of dietary supplementation with SAqFSC-P, PAqFSC-P, or bicyclol on serum and hepatic GLU contents in normal and hypercholesterolemic mice.** Mice were fed with ND or HCBD without and with supplementation with SAqFSC-P or PAqFSC-P, or BL at the indicated concentration for 10 days. Experimental details were described in Figure 1. Serum (Figure **A**) and hepatic (Figure **B**) glucose (GLU) levels were measured. Value given are the means ± S.E.M., with n = 10. ^**^P < 0.01 vs. control ND; ^††^*P* < 0.01 vs. control HCBD, using a one-way ANOVA followed by Dunnett’s multiple comparisons test or post-hoc analysis.

While supplementation with SAqFSC-P or PAqFSC-P at a dose of 1% and 9% did not affect the serum GLU levels in ND- and HCBD-fed mice, HCBD feeding significantly decreased serum GLU level by 11% (Figure 3A). SAqFSC-P or PAqFSC-P supplementation at doses of 1% and 9% decreased hepatic GLU level (by 16 - 31%) in ND-fed mice. HCBD feeding increased hepatic GLU level by 19%, when compared with that of mice fed with ND. Supplementation with SAqFSC-P or PAqFSC-P at 1% and 3% decreased hepatic GLU level (by 12 - 18%) in HCBD-fed mice (Figure 3B). While bicyclol supplementation produced no detectable changes in serum GLU levels in both ND- and HCBD-fed mice, it decreased hepatic GLU level (by 32%) in ND-fed but not HCBD-fed mice.

### Hepatic index/protein/function

Supplementation with PAqFSC-P at 9% increased hepatic index by 9% in ND-fed mice, but not in HCBD-fed mice. PAqFSC-P supplementation at 1 or 9% increased hepatic protein content by 6 and 12% in ND-fed mice, respectively. However, only 1% SAqFSC-P supplementation decreased hepatic protein level by 7% in hypercholesterolemic mice, when compared with the un-supplemented HCBD-fed mice. Bicyclol treatment increased hepatic protein by 11% in ND-fed mice. SAqFSC-P and bicyclol supplementation suppressed serum ALT activity by 24 and 29%, respectively, in ND-fed mice. Bicyclol also inhibited ALT activity by 42% in HCBD-fed mice. HCBD feeding increased hepatic weight, hepatic protein content, and ALT activity (by 12, 13 and 38%, respectively) (Table 2).

**Table 2:** Effects of dietary supplementation with SAqFSC-P, PAqFSC-P, or bicyclol on hepatic index, protein and function in normal and hypercholesterolemic mice

**Groups**	**Drug concentration (%, w/w)**	**Hepatic index (%)**	**Hepatic protein (g/g liver tissue)**	**Serum ALT activity (U/L)**
***Normal mice***				
Control ND	-	6.59 ± 0.10	0.138 ± 0.003	36.50 ± 3.61
SAqFSC-P/ND	1	6.76 ± 0.13	0.131 ± 0.003	27.60 ± 0.72^**a**^
	9	6.91 ± 0.13	0.140 ± 0.004	30.33 ± 2.41
PAqFSC-P/ND	1	6.73 ± 0.11	0.146 ± 0.002^**a**^	32.80 ± 1.63
	9	7.16 ± 0.17^**b**^	0.155 ± 0.003^**b**^	32.00 ± 1.81
Bicyclol/ND	0.025	6.69 ± 0.11	0.153 ± 0.002^**b**^	26.00 ± 1.67^**a**^
***Hypercholesterolemic mice***				
Control ND	-	6.43 ± 0.77	0.133 ± 0.004	36.18 ± 2.17
Control HCBD	-	7.19 ± 0.13^**b**^	0.150 ± 0.002^**a**^	50.09 ± 5.81^**a**^
SAqFSC-P/HCBD	1	7.17 ± 0.11	0.140 ± 0.005^**c**^	47.26 ± 3.58
	3	7.06 ± 0.16	0.153 ± 0.003	46.32 ± 2.52
PAqFSC-P/HCBD	1	6.99 ± 0.16	0.147 ± 0.004	47.31 ± 3.38
	3	7.04 ± 0.13	0.156 ± 0.003	43.60 ± 3.90
Bicyclol/HCBD	0.025	7.25 ± 0.06	0.149 ± 0.003	29.30 ± 1.52^**c**^

Experimental details were described in Figure 1 and Table 1. Mice were fed with ND or HCBD containing SAqFSC-P, PAqFSC-P, or bicyclol at the indicated concentration for 10 days. Hepatic index (liver weight/body weight × 100), protein (use Bradford assay) and function indicated by alanine aminotransferase (ALT) were then measured. Mouse model of hypercholesterolemia was generated by HCBD. Values given are the means ± S.E.M., with n = 10. ^**a**^*P* < 0.05, ^**b**^*P* < 0.01 vs. control ND; ^**c**^*P* < 0.05 vs. control HCBD, using a one-way ANOVA follow by Dunnett’s multiple comparisons test or post-hoc analysis.

### Fatty index

**Figure 4:**
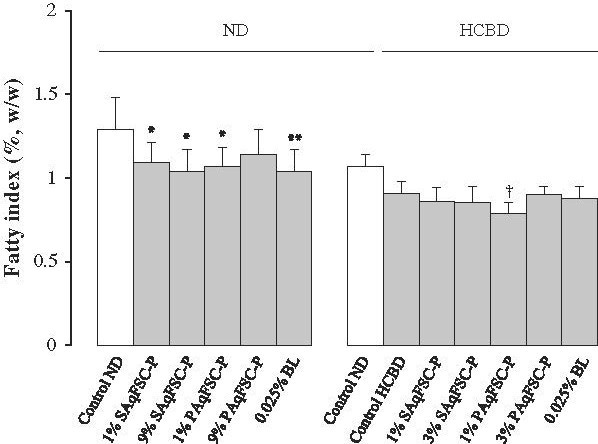
**Effects of dietary supplementation with SAqFSC-P, PAqFSC-P, or bicyclol on fatty index in normal and hypercholesterolemic mice**. Mice were fed with ND or HCBD without and with supplementation with SAqFSC-P, PAqFSC-P, or BL at the indicated concentration for 10 days. Experimental details were described in Figure 1. Fatty index was estimated from the ratio of epididymal fat weight to body weight (epididymal fat weight/body weight × 100). Value given are the means ± S.E.M., with n = 10. ^*^P < 0.05, ^**^P < 0.01 vs. control ND; ^†^*P* < 0.05 vs. control HCBD, using a one-way ANOVA followed by Dunnett’s multiple comparisons test or post-hoc analysis.

Supplementation with SAqFSC-P at 1% and 9% or PAqFSC-P at 1% decreased the fatty index (by 15 and 18% or 16%, respectively) in ND-fed mice. HCBD feeding did not cause any detectable change in fatty index in mice, and both SAqFSC-P and PAqFSC-P supplementation did not produce any changes in fatty index in HCBD-fed mice, except for 1% PAqFSC-P supplementation which caused a slight but significant decrease (by 13%) in fatty index. While bicyclol supplementation at 0.025% reduced the fatty index (by 19%) in ND-fed mice, it did not cause any effect on HCBD-fed mice (Figure 4).

### Body weight gain and food/drug intake

Supplementation with SAqFSC-P at 1% or 9% decreased the body weight gain during the period from D5 to D10 (by 23% and 21%, respectively) in ND-fed mice. Mice fed with HCBD showed a significant decrease in body weight gain from D1 to D5 (by 6%). The daily intake of SAqFSC-P or PAqFSC-P was estimated to be 1.4 - 1.5 g/kg (based on an equivalent amount of crude herb) at 1% supplementation, 4.5 - 4.7 g/kg at 3% supplementation in HCBD-fed mice and 13.6 - 14.2 g/kg/day at 9% supplementation in ND-fed mice. Bicyclol supplementation decreased the body weight gain from D5 to D10 (by 17%) in ND-fed but not HCBD-fed mice. The daily intake of bicyclol was estimated to be 0.04 g/kg (Table 3). SAqFSC-P and bicyclol supplementation did not reduce the body weight gain from D1 to D10.

**Table 3:** Effects of dietary supplementation with SAqFSC-P, PAqFSC-P, or bicyclol on body weight gain and food/drug intake in normal and hypercholesterolemic mice

**Groups**	**Drug concentration (%, w/w)**	**Body weight gain (g)**		**Food intake (g/kg/day)**	**Drug intake (g/kg/day)**
		**D 1 → D 5**	**D 5 → D 10**		
***Normal mice***					
Control ND	-	7.41 ± 0.29	4.46 ± 0.22	156.4	-
SAqFSC-P/ND	1	7.52 ± 0.07	3.41 ± 0.18^**a**^	142.2	1.42
	9	7.19 ± 0.16	3.53 ± 0.27^**a**^	157.5	14.18
PAqFSC-P/ND	1	7.42 ± 0.17	3.99 ± 0.36	143.7	1.44
	9	7.49 ± 0.35	4.54 ± 0.26	151.4	13.63
Bicyclol/ND	0.025	7.41 ± 0.20	3.70 ± 0.26^**a**^	158.3	0.04
***Hypercholesterolemic mice***					
Control ND	-	10.42 ± 0.25	3.04 ± 0.14	162.7	-
Control HCBD	-	9.76 ± 0.16^**a**^	2.81 ± 0.12	151.0	-
SAqFSC-P/HCBD	1	9.71 ± 0.29	3.02 ± 0.10	151.4	1.51
	3	9.36 ± 0.20	2.98 ± 0.19	150.0	4.50
PAqFSC-P/HCBD	1	10.02 ± 0.28	2.78 ± 0.11	150.5	1.51
	3	9.66 ± 0.15	3.11 ± 0.16	157.5	4.72
Bicyclol/HCBD	0.025	10.36 ± 0.23	2.72 ± 0.13	152.5	0.04

Experimental details were described in Figure 1. The dosages (g/kg/day, based on crude herbal material) were determined with respect to the amount of ingested diet (g/day/kg) and drug concentration in the diet. Values given are the means ± S.E.M., with n = 10. ^**a**^*P* < 0.05 vs. control ND, using a one-way ANOVA followed by Dunnett’s multiple comparisons test or post-hoc analysis.

## Discussion

Hypercholesterolemia, which is a sub-type of hyperlipidemia characterized by significant elevation of serum TC level, is causally related to the pathogenesis of CHD, NAFLD, atherosclerosis, diabetes and metabolic syndrome [20,21]. Unhealthy eating habit is one major cause of hypercholesterolemia in humans. In this study, except for serum TG level, both serum and hepatic lipid levels were found to be increased by feeding HCBD in mice. Unlike other atherogenic diet containing cholesterol, sucrose, hydrogenated vegetable oil, sodium cholate, lactose, choline chloride, and thiouracil [22], which elevated serum TG and TC levels, HCBD used in this study did not increase serum TG levels. FSC-P (ie. SAqFSC-P and PAqFSC-P) intake for 10 days markedly reduced hepatic TC, TG, and GLU contents, as well as serum TG levels in normal and hypercholesterolemic mice, as compared with the respective control mice, but it produced no significant effect on serum TC level. This observation suggested that the lipid-lowering effect of FSC-P may depend on basal lipid levels in the body. Our previous study has shown that schisandrin B, a major active ingredient presented in pulp fraction of FSC, lowered fat accumulation in L-02 cells incubated with free fat acid via inhibition of ADRP and SREBP-1 expression [23]. This might also explain the hepatic lipid-lowering effects of SAqFSC-P and PAqFSC-P supplementation. It has been demonstrated that FSC pulp mainly contains polysaccharides/sugars and organic acids, as well as dibenzocyclooctadiene lignans such as schisandrins (A, B, and C) and gomisins (A and N), which are believed to be the active ingredients in FSC [24,25]. In addition, several other dibenzocyclooctadiene lignans (Schisandra red pigment) have been isolated from the fruits of Schisandra wilsoniana [26,27].

LDL in the blood is originated from very low density-LDL (VLDL) produced in the liver in association with apoprotein B-100. Being associated with an increased risk of atherosclerosis and CHD, LDL is also called “bad cholesterol” [28,29]. In contrast, HDL, which is protective in nature, is called “good cholesterol”. Hypercholesterolemia, including familial hypercholesterolemia, in human typically shows high levels of LDL and lower levels of HDL. In the current study, although the serum HDL level was not reduced, the LDL level was markedly elevated in hypercholesterolemic mice. Furthermore, LDL/HDL and N-HDL values were increased, but HDL/LDL values were decreased in mice fed with HCBD-fed mice. As the predictive indicator of atherosclerosis and coronary artery calcium [30], high level of LDL and N-HDL caused by HCBD may hasten the development of hypercholesterolemia. In addition, the accumulation of fat in hepatic tissue may lead to liver damage in HCBD-fed mice, as indicated by an increase in serum ALT activity. FSC-P did not affect serum HDL and LDL levels in both normal and hypercholesterolemic mice, but PAqFSC-P decreased N-HDL values in HCBD-fed mice. N-HDL cholesterol can predict the risk of CHD [31] and serves as a readily available parameter for estimating the risk of cognitive impairment in type 2 diabetic [32]. In this regard, PAqFSC-P may be used for the prevention of hypercholesterolemia related CHD and memory disorders in patients with diabetes mellitus.

It has been well-known that lipid/fat metabolism has a close relationship with GLU metabolism. The relevant metabolic disorders constitute the pathological basis of modern epidemics such as hyperlipidemia, hyperglycemia, and hypertension, as well as metabolic syndrome, including obesity. In this study, HCBD-feeding was found to elevate serum TC and hepatic TC, TG, and GLU, as well as reduce serum GLU in mice. As such, the GLU metabolism is altered by hypercholesterolemic conditions. Studies have demonstrated that insulin resistance is the pathophysiological hallmark of NAFLD [33,34]. The suppression of insulin sensitivity results in the accumulation of GLU/fat in the liver, and then leading to the development of NAFLD. As for HCBD-induced hypoglycemia may be an event secondary to the elevation in hepatic GLU contents. It is possible that release of hepatic GLU is inhibited in the HCBD-fed mice. Low hepatic GLU and lipid levels caused by FSC-P supplementation might result from the FSC-P-induced increase in hepatic insulin sensitivity in HCBD-fed mice [35,36]. FSC-P supplementation decreased the epididymal fat mass in mice fed with ND. However, due to the lowered fat index in HCBD-fed mice, FSC-P-induced fat loss was not observed in hypercholesterolemic mice. On the other hand, body weight loss was observed during D5 and D10 in ND-fed mice supplemented with SAqFSC-P/ND. The mechanism underlying the FSC-P-induced loss in body weight/fat and hepatic lipid may also involve the inhibition of ADRP and SREBP-1 expression [23].

Previous studies have demonstrated that FSC related active compounds such as bicyclol, bifendate, and schisandrin B, when administered at high doses, can elevate serum TG level and hepatic weight in mice. As such, we have established new animal models of hyperlipidemia using schisandrin B [37-39] and bifendate [40]. However, FSC-P extracts used in the present study only produced minimal effect on serum TG level and hepatic weight. FSC-P at a high dose (9%, up to 14 g/kg/day for 10 days) did not affect animal behaviors (data not shown), which indicated the safety of FSC-P intake on central nervous system. In addition, serum creatinine levels, indicative of renal function, were not changed after FSC-P consumption (data not shown). Although we were not able to show a dose–response relationship in certain pharmacological activities of FSC-P, it was evident that FSC-P could significantly influence the hepatic lipid profile in hypercholesterolemic mice. Owing to the fact that chemical components in an herbal extract produce a wide spectrum of biological actions, the possible interactions among them may preclude the display of a dose-dependent relationship of a pharmacological activity. In the present study, some effects produced by SAqFSC-P or PAqFSC-P at high dose (3% or 9%) were less significant than at low dose (1%). Therefore, caution has to be exercised in determining the dosage of FSC administration in clinical situations.

Despite the fact that biochemical mechanisms underlying the development of hyperlipidemia and its complications are widely investigated, hyperlipidemia remains the major cause of morbidity and mortality throughout the world. While the clinical management of hyperlipidemia has yet to be optimized, Chinese herbs, which have been used for thousands of years, may serve as an alternative treatment for hyperlipidemia. In the present study, the effect of FSC-P was investigated in the mouse model of hypercholesterolemia. Although both SAqFSC-P and PAqFSC-P supplementations notably lowered hepatic TG and GLU contents and elevated hepatic TC levels in ND-fed mice, no detectable changes were observed in serum lipid and apolipoprotein levels. This finding suggests that the lipid-modulating effect of FSC-P may be influenced by factors such as lipid levels in tissues. However, it is still not clear whether dietary FSC-P-induced increase in hepatic TC content is good or bad in ND-fed mice. Both SAqFSC-P and PAqFSC-P supplementations decreased serum TG levels and hepatic TC/TG contents in HCBD-fed mice, but no detectable effect on serum TC level was observed.

In conclusion, FSC pulp (i.e. SAqFSC-P or PAqFSC-P) supplementation ameliorated the fatty liver condition in HCBD-fed mice. FSC pulp supplementation decreased hepatic GLU and serum TG levels in both ND- and HCBD-fed mice. However, the supplementation with bicyclol did not affect serum and hepatic lipid levels in HCBD-fed mice (summarized in Table 4). SAqFSC-P and PAqFSC-P produce the similar beneficial outcomes in hypercholesterolemic mice. Naturally-occurring ingredients from FSC pulp may provide a safe alternative for the management of fatty liver and/or lipid disorders in humans.

**Table 4:** A summary of the current study

**Groups**	**Dietary SAqFSC-P supplement**			**Dietary PAqFSC-P supplement**			**Bicyclol**
	**1%**	**3%**	**9%**	**1%**	**3%**	**9%**	**0.02 5%**
***Normal mice***							
Serum TC	-		-	-		-	-
TG	-		-	↓		-	↓
HDL	-		-	-		-	-
LDL	-		-	-		-	-
N-HDL	-		-	-		-	-
Glucose	-		-	-		-	-
ALT activity	↓		-	-		-	↓
Hepatic TC	↑		↑	↑		↑	-
TG	↓		↓	-		-	↓
Glucose	↓		↓	↓		↓	↓
Index	-		-	-		↑	-
Protein	-		-	↑		↑	↑
Fat index	↓		↓	↓		-	↓
Body weight gain	↓		↓	-		-	↓
***Hypercholesterolemic mice*** (change vs. normal)							
Serum TC (↑)	-	↓		-	-		-
TG (-)	↓	-		↓	↓		-
HDL (-)	-	-		-	-		-
LDL (↑)	-	-		-	-		-
N-HDL (↑)	-	-		↓	↓		-
Glucose (↓)	-	-		-	-		-
ALT activity (↑)	-	-		-	-		↓
Hepatic TC (↑)	↓	↓		↓	-		-
TG (↑)	↓	↓		↓	↓		-
Glucose (↑)	↓	↓		↓	↓		-
Protein (↑)	↓	-		-	-		-
Index (↑)	-	-		-	-		-
Fat index (-)	-	-		↓	-		-
Body weight gain (↓)	-	-		-	-		-

TC: total cholesterol; TG: triglyceride; HDL: high-density lipoprotein; LDL: low-density lipoprotein; N-HDL: non-HDL; ALT: alanine aminotransferase.

↑: increased or elevated; ↓: decreased or inhibited; -: unaltered.

## Materials and methods

### Herbal material and extraction procedure

**Figure 5:**
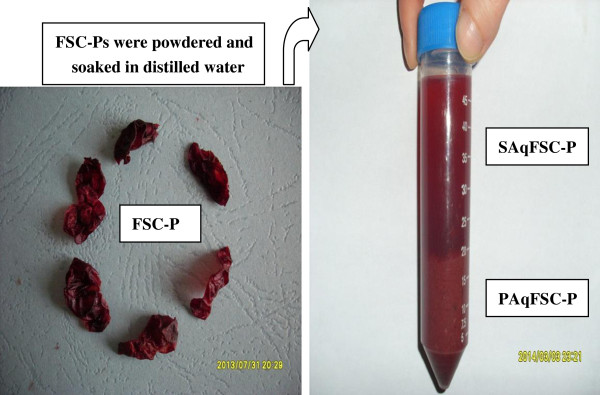
**Preparation of SAqFSC-P and PAqFSC-P.** FSC-P: *Fructus Schisandrae Chinensis* pulp; SAqFSC-P: supernatant of aqueous extract of FSC-P; PAqFSC-P: precipitate of aqueous extract of FSC-P.

FSC, the fruit of *Schisandra Chinensis* (Turcz.) Baill., was purchased from the Anguo Chinese herbs market and authenticated by professor Chun-Sheng Liu in the Beijing University of Chinese Medicine. The fruit pulp and seed were manually separated, then dried at room temperature. The weight of pulp and seed was 63% and 37% of total weight, respectively. FSC pulp (300 g) were powdered and soaked in 300 mL distilled water for 4 h at room temperature (approximately 25°C), and they were then washed five times with 300 mL distilled water. The pooled aqueous extract was centrifuged at 2000 × *g* for 3 min. The supernatant was concentrated under reduced pressure by rota-evaporation at 50°C to obtain 200 mL of SAqFSC-P (i.e., 0.95 g (dry weight) of FSC-P equivalent for every 1 mL of SAqFSC-P. As for the sediment, every g (wet weight) of PAqFSC-P was equivalent to 2.3 g of dried FSC-P. Both SAqFSC-P and PAqFSC-P were stored at 4°C until use (Figure 5).

### Chemicals and reagents

Cholesterol (certificate no. 041103) and bile salt (certificate no. 000710) were obtained from Beijing Chemical Reagent Co. (Beijing, China). Fenofibrate (certificate no. 0405030) was bought from Beijing Yongkang Medical Ltd. (Beijing, China). Bicyclol was purchased from Beijing Union Pharm. (Beijing, China). Assay kits for TC (certificate no. 121471), TG (certificate no. 135991) and GLU (certificate no. 122991) were bought from Zhongsheng Beikong Bio-technology and Science Inc. (Beijing, China). Assay kits for HDL and LDL levels, as well as alanine aminotransferase (ALT) were obtained from Zhongsheng Beikong Bio-technology and Science Inc. (Beijing, China) or Beijing Leadman Biochemistry Co., Ltd. (Beijing, China). Coomassie brilliant blue G250 (certificate no. 20130624) was purchased from Nanjing Jiancheng Bioengineering Institute (Nanjing, China).

### Animal treatment

Male ICR mice [Grade II, certificate No. SCXK (jing) 2006–0009], weighing 17–20 g (two-month-old), and the standard mouse chow diet were supplied by Vital River Lab Animal Co. Ltd. (Beijing, China). All animals were maintained on a 12 h (light on 700–1900 h) light–dark cycle at 20–21°C, with a relative humidity of 50 - 55%. They were allowed free access to water and food. Blood and liver/fat tissue samples were obtained from ether-anesthetized animals which had been fasted for 6 h (from 600 to 1200). All experimental procedures were approved by the University Committee on Research Practice in Beijing University of Chinese Medicine.

### Study design 1: normal diet (ND)-fed mice

This study was aimed to examine whether the oral administration of FSC-P could produce any toxic effects in mice fed with ND. Mice were divided into 6 groups of 10 animals in each: (1) control mice fed with ND; (2) and (3) mice fed with ND supplemented with 1% and 9% (w/w) SAqFSC-P, respectively; (4) and (5) mice fed with ND supplemented with 1% and 9% PAqFSC-P, respectively; and (6) mice fed ND supplemented with 0.025% bicyclol. Currently, bicyclol (a synthetic derivative of dibenzocyclooctadiene lignan from FSC) is used to treat hepatitis in China. Therefore, bicyclol was selected as a positive control. Mice were sacrificed ten days after the drug treatment.

### Study design 2: High cholesterol/bile salt diet (HCBD)-fed mice

This study was aimed to investigate whether the oral administration of FSC-P could produce any beneficial effect on mice fed with HCBD containing cholesterol/bile salt (1%/0.3%, w/w). Seventy mice were randomly assigned to seven groups of 10 animals in each: (1) control mice fed with ND; (2) control (ie. untreated) mice fed with HCBD; (3)-(6) mice fed with HCBD supplemented with SAqFSC-P or PAqFSC-P at 1% and 3% (w/w); (7) mice fed with HCBD supplemented with bicyclol at 0.025%. Ten days later mice were killed and biochemical analyses were performed. Our previous studies showed that high levels of serum and hepatic lipids were observed in mice after 10 days on high-fat diet [15-19].

### Biochemical analysis

Serum and liver samples were obtained 24 h after the last day of the experiment. Serum samples were prepared by centrifuging the whole blood obtained from the orbital vein for 8 min at 2000 × *g* and stored at -70°C until use for biochemical analyses. Liver tissue samples were homogenized in 9 volumes of saline solution by two 10-s bursts of a tissue disintegrator at 13,500 rpm. The homogenates were then centrifuged at 2000 × *g* for 15 min to obtain the supernatants. Thirty μL of the hepatic supernatant and 10 μL serum was used to determine TG and TC levels with GPO-PAP and COD-PAP method, respectively. Ten μL serum and 5 μL hepatic supernatant were used to determine the GLU levels with GOD-POD method. One hundred μL of hepatic supernatant were used to measure protein level using Bradford assay. Assays were performed by using assay kits according to the manufacturer’s instructions. HDL/LDL, LDL/HDL and N-HDL values were estimated by calculating the ratio of HDL to LDL and LDL to HDL, and the difference between TC and HDL, respectively. ALT activity was measured by automatic Biochemistry Analyzer (Beckman coulter Synchron CX4 PRO, Brea, CA, USA).

### Measurement of hepatic index and fatty index

Liver weight was measured and hepatic index was estimated from the ratio of liver weight to body weight (liver weight/body weight × 100). Epididymal fat weight was measured and fatty index was estimated from the ratio of fat weight to body weight (epididymal fat weight/body weight × 100).

### Statistical analysis

Data were expressed as means ± S.E.M., and they were analyzed by One-way ANOVA followed by Dunnett’s multiple comparison test or post-hoc analysis using SPSS 16.0 statistical analysis program. *P* < 0.05 was considered as significant.

## Abbreviations

NAFLD: Nonalcoholic fatty liver disease; FSC: *Fructus Schisandrae Chinensis*; SAqFSC-P: Supernatant of aqueous extract of FSC pulp; PAqFSC-P: Precipitate of aqueous extract of FSC pulp; TG: Triglyceride; TC: Total cholesterol; HDL: High-density lipoprotein cholesterol; LDL: Low-density lipoprotein cholesterol; GLU: Glucose; ALT: Alanine aminotransferase; CHD: Coronary heart disease.

## Competing interests

The authors declare that they have no competing interests.

## Authors’ contributions

Design of the study: SYP, ZLY; conduct of the study: NS, YZ, PLZ, XYW, ZSC; data collection: XYW, NS; data analysis: SYP, SFZ; data interpretation: SYP, KMK; manuscript writing: NS, SYP, KMK. All authors read and approved the final manuscript.

## Authors’ information

Si-Yuan Pan is a professor; Yi Zhang and Xiao-Yan Wang are Doctor Degree Graduate; Nan Sun, Zhu-Sheng Chu, and Pei-Li Zhu are Master Degree Graduate; Zhi-Ling Yu, PhD, is an associate professor; Shu-Feng Zhou, PhD and Kam-Ming Ko, PhD, are professors.
